# Supplementary material for: Bioenergetic and Proteomic Profiling of ARID1A-Deficient Ovarian Clear Cell Carcinoma under Metabolic Challenge
Source: bioRxiv. 2026 Oct 16:2026.10.09.757928. Preprint. [Version 1] doi: 10.64898/2026.10.09.757928 (PMC13726901; doi:10.64898/2026.10.09.757928)
Supplement: Supplementary file 1 [file media-1.pdf]

Supporting Information

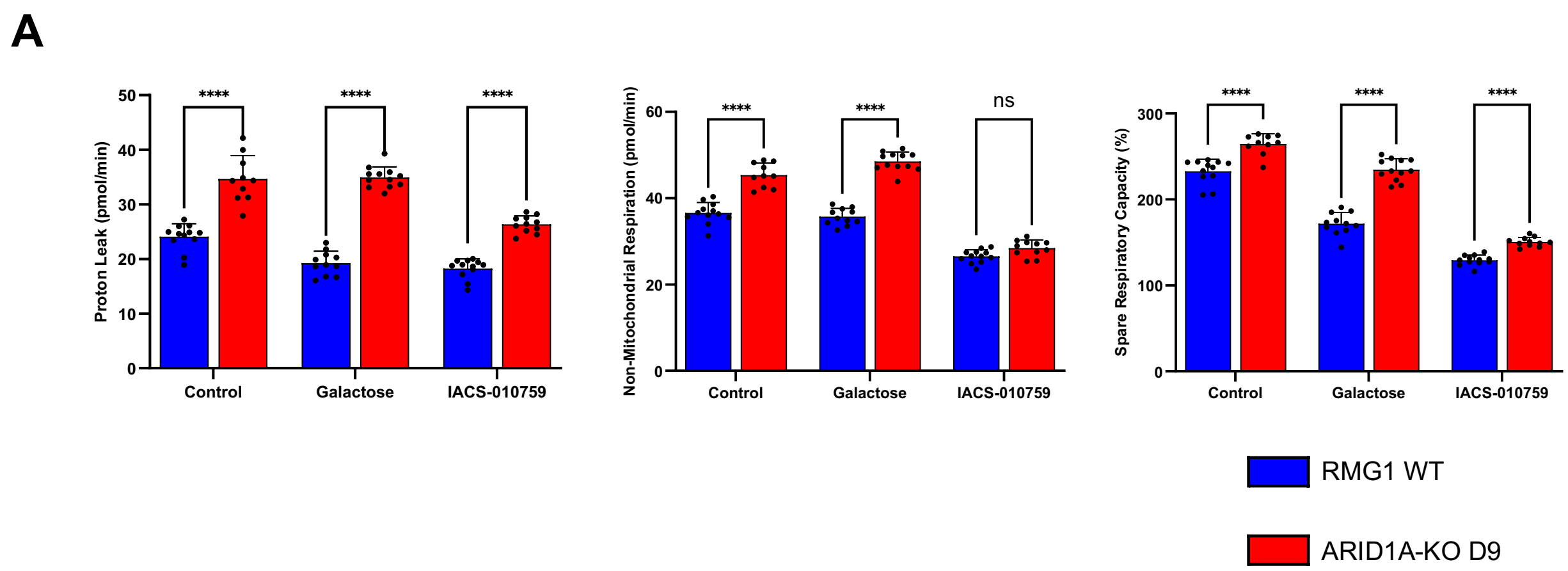

Figure S1. Additional mitochondrial respiratory parameters in RMG1 WT and D9 cells. (A) Proton leak, non-mitochondrial respiration, and spare respiratory capacity expressed as a percentage of basal respiration in RMG1 WT and ARID1A-knockout D9 cells under the indicated metabolic conditions. Data points represent individual Seahorse wells and bars represent mean ± SEM.

Sample	Range of biological replicate protein abundance correlations (R)
WT Ctrl	0.992 – 0.993
D9 Ctrl	0.994 – 0.996
WT Gal	0.994 – 0.995
D9 Gal	0.987 – 0.991
WT C1	0.989 – 0.994
D9 C1	0.993 – 0.995

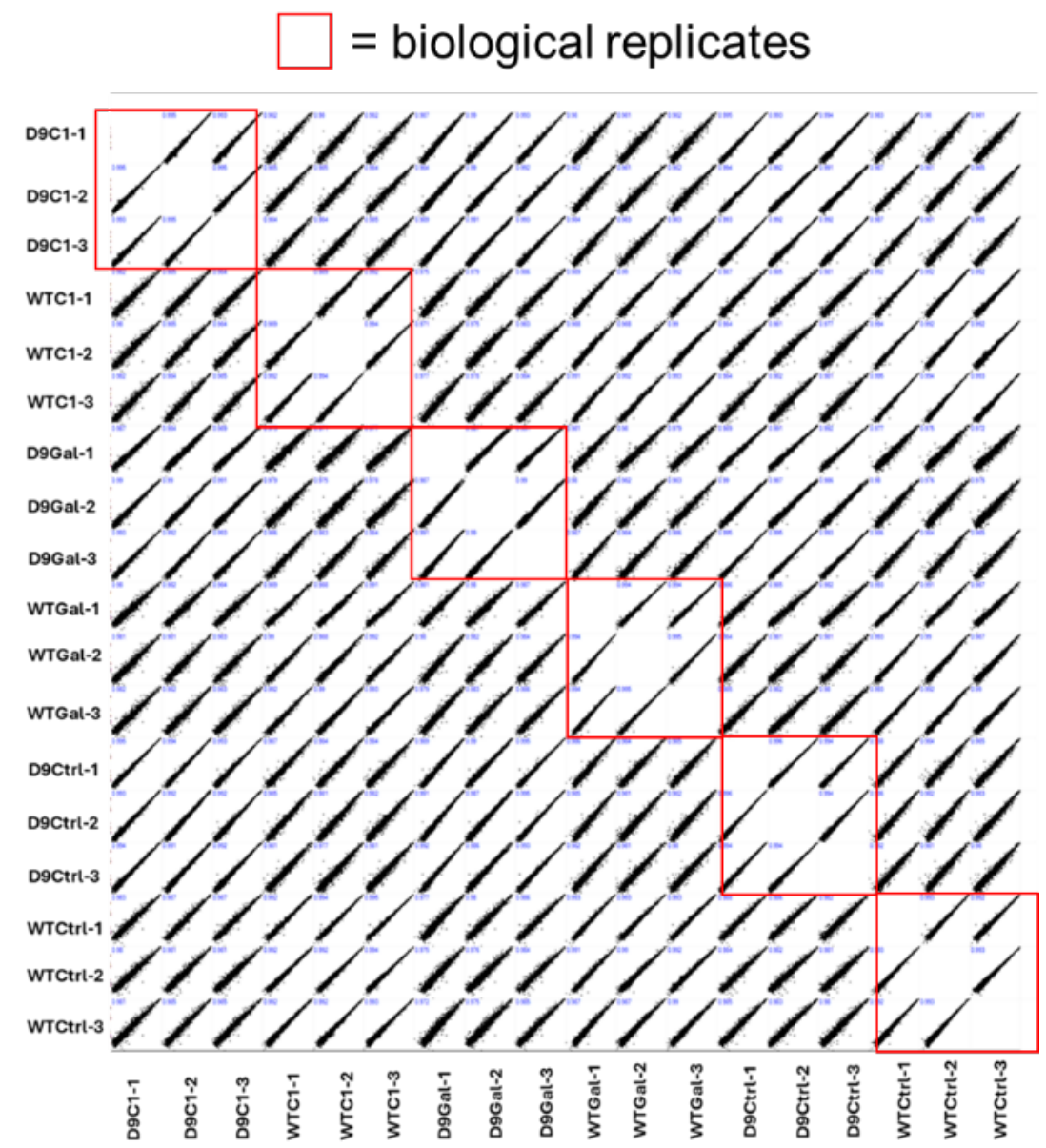

Figure S2. Correlation plots of protein abundances across all samples. R = Pearson correlation. n = 3 biological replicates per condition.

Complex I proteins

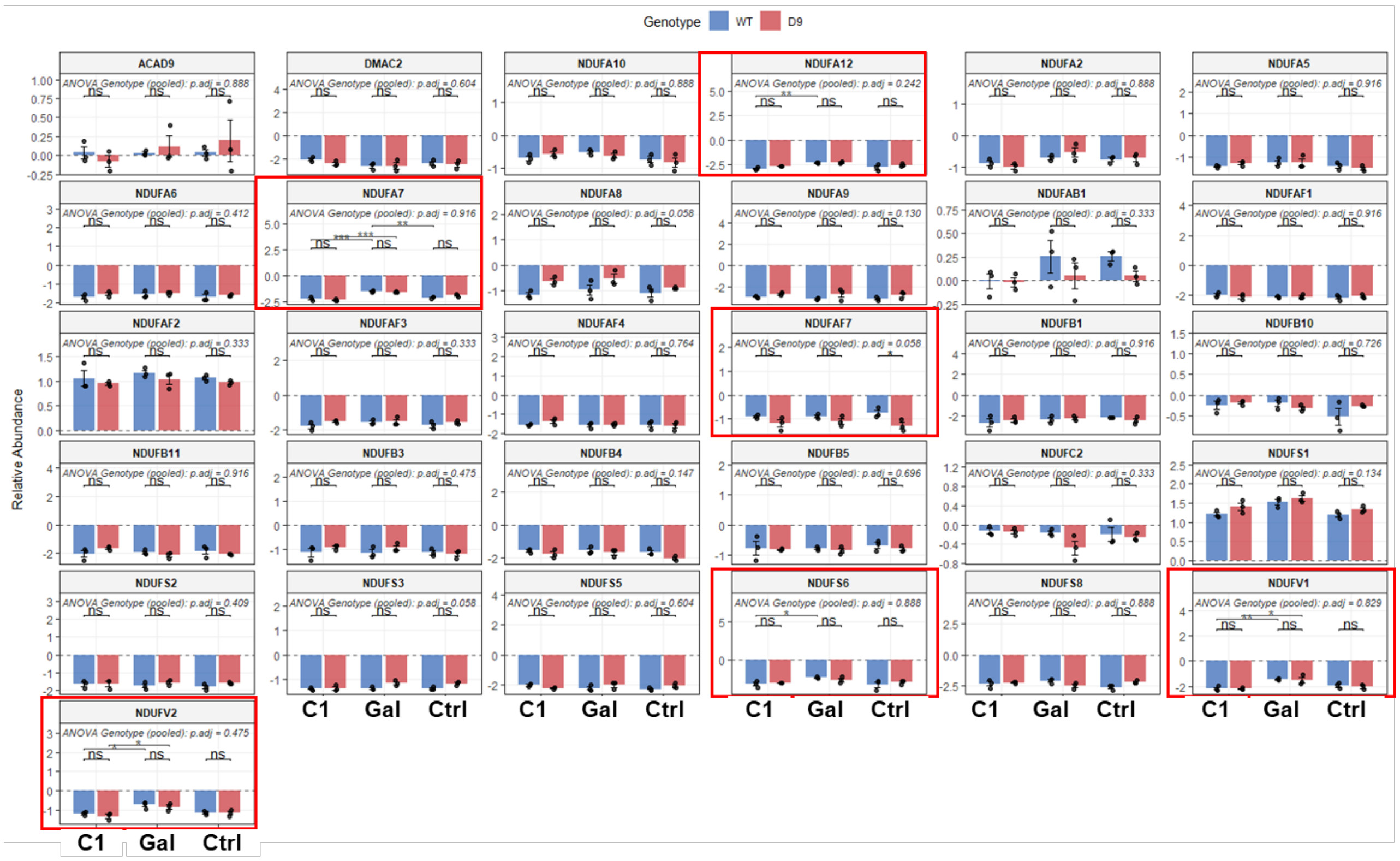

Figure S3. Pairwise comparisons of Complex I protein abundances. Red boxes indicate proteins with significantly different protein abundances between genotypes or metabolic conditions. Limma-moderated t-tests with Benjamini Hochberg-adjusted FDR. **p*.adj<0.05, ***p*.adj<0.01, ****p*.adj<0.001.

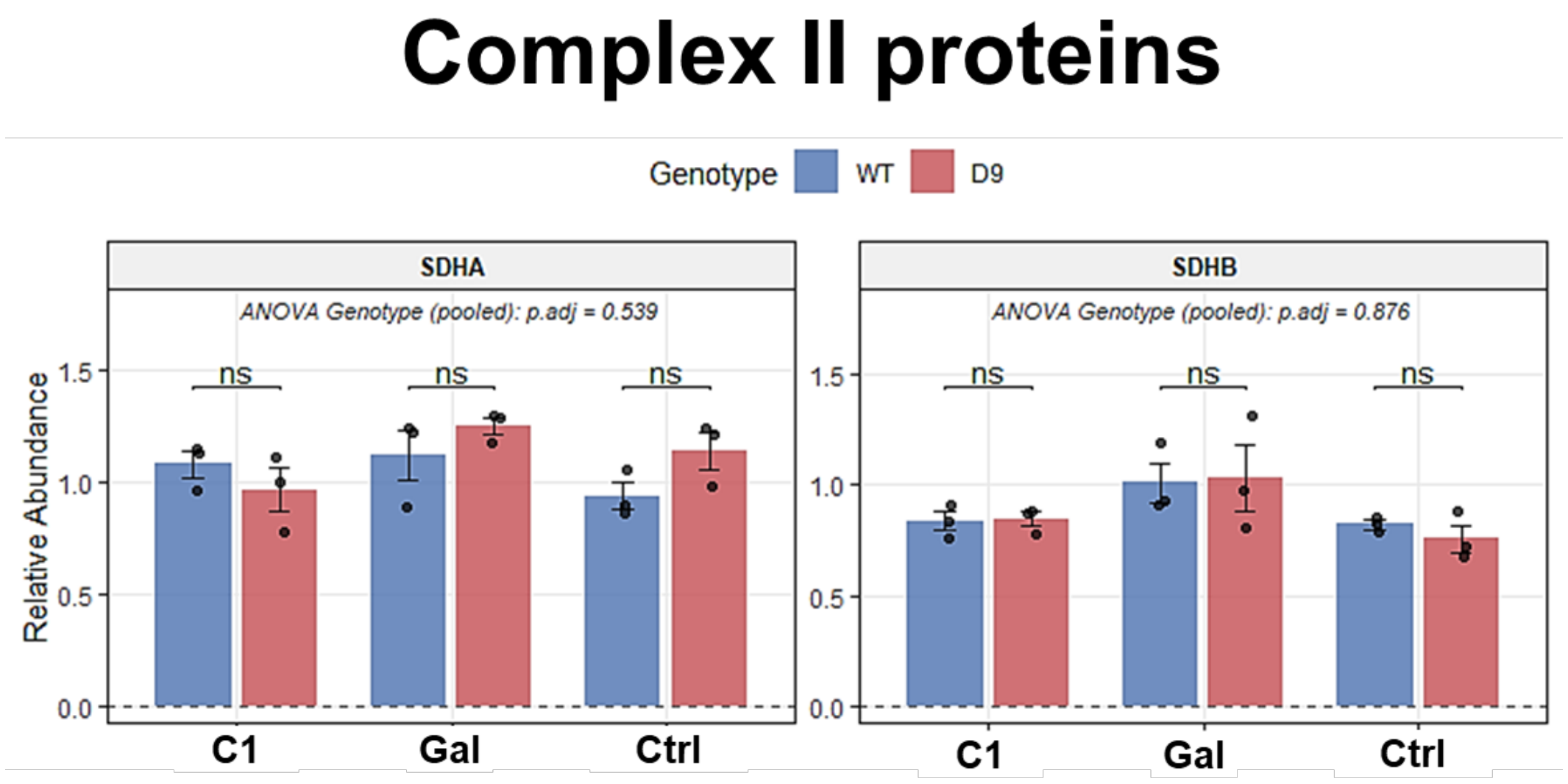

Figure S4. Pairwise comparisons of Complex II protein abundances. Limma-moderated t-tests with Benjamini Hochberg-adjusted FDR. **p*.adj<0.05, ***p*.adj<0.01, ****p*.adj<0.001.

Complex III proteins

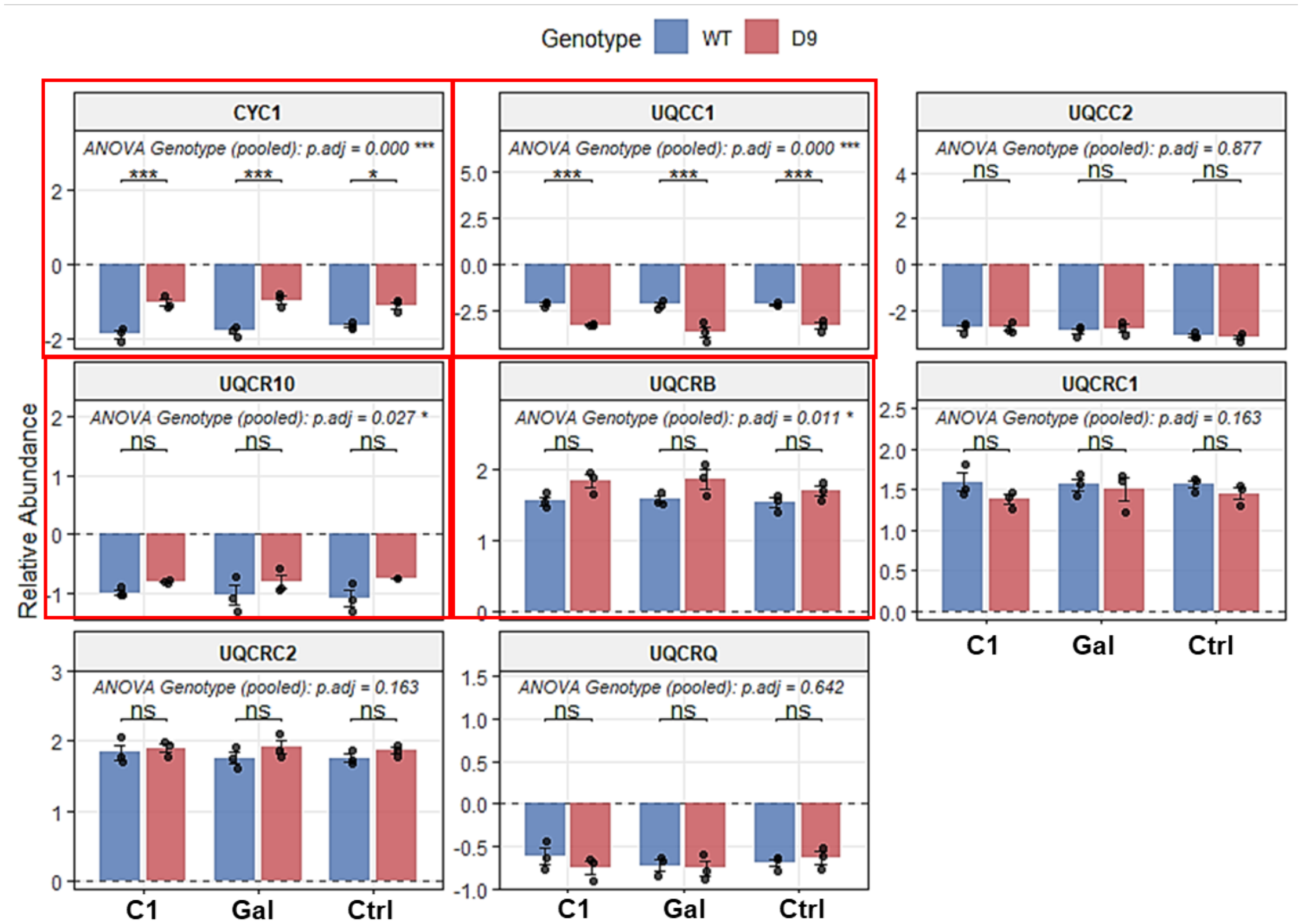

Figure S5. Pairwise comparisons of Complex III protein abundances. Red boxes indicate proteins with significantly different protein abundances between genotypes or metabolic conditions. Limma-moderated t-tests with Benjamini Hochberg-adjusted FDR. *p.adj<0.05, **p.adj<0.01, ***p.adj<0.001.

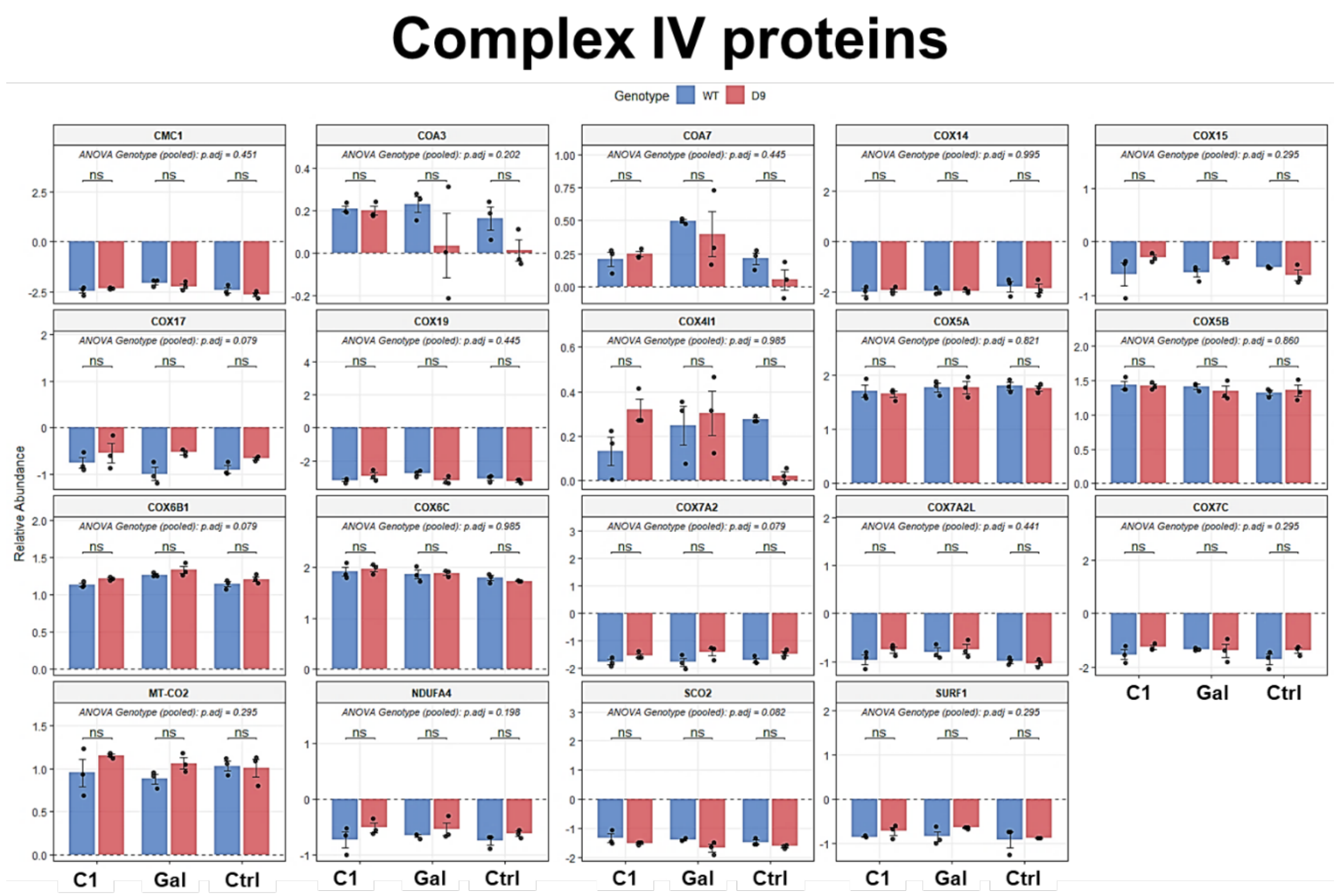

Figure S6. Pairwise comparisons of Complex IV protein abundances. Limma-moderated t-tests with Benjamini Hochberg-adjusted FDR. **p*.adj<0.05, ***p*.adj<0.01, ****p*.adj<0.001.

Complex V proteins

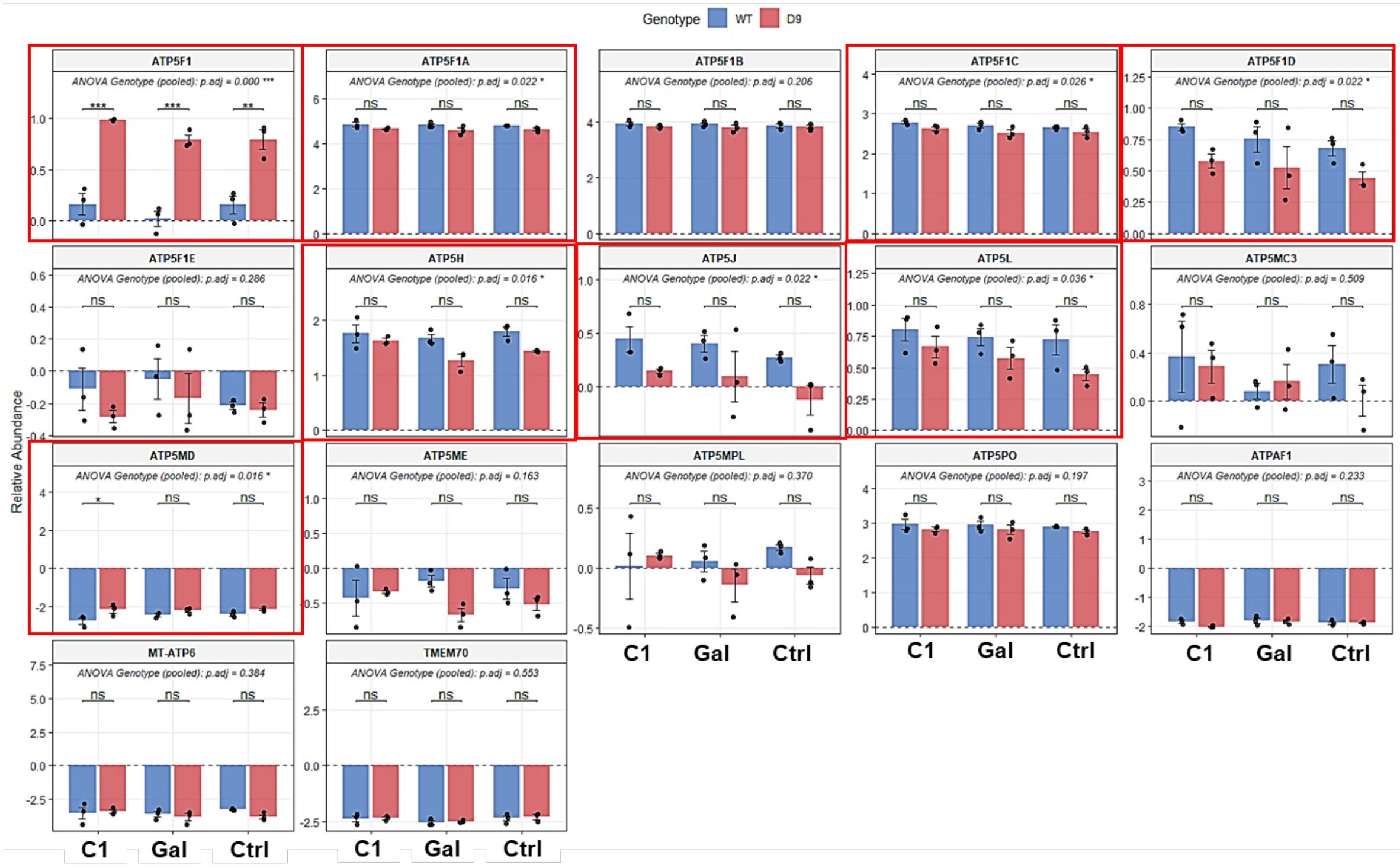

Figure S7. Pairwise comparisons of Complex V protein abundances. Red boxes indicate proteins with significantly different protein abundances between genotypes or metabolic conditions. Limma-moderated t-tests with Benjamini Hochberg-adjusted FDR. **p*.adj<0.05, ***p*.adj<0.01, ****p*.adj<0.001.
